# Metastatic Melanoma Presenting as a Ventricular Arrhythmia

**DOI:** 10.7759/cureus.7634

**Published:** 2020-04-11

**Authors:** Arslan Babar, Hassan Lak, Sanchit Chawla, Gauranga Mahalwar, Anjli Maroo

**Affiliations:** 1 Internal Medicine, Cleveland Clinic - Fairview Hospital, Cleveland, USA; 2 Internal Medicine, Cleveland Clinic Akron General, Akron, USA; 3 Cardiology, Cleveland Clinic - Fairview Hospital, Cleveland, USA

**Keywords:** melanoma, arrhythmia, ventricular tachycardia

## Abstract

Melanoma is a highly aggressive disease with the risk of developing metastasis to virtually all organs including the heart, which can manifest as arrhythmia, right ventricular obstruction, heart failure, or pericardial effusion. Only a few reports are found in the literature of metastatic melanoma, causing ventricular arrhythmia. Prior to the advent of contemporary therapies, cardiac metastases implied a very poor prognosis. The use of immune checkpoint inhibitors and targeted therapy has greatly improved survival outcomes of metastatic melanoma. Aggressive therapy of cardiac metastasis including cardiac surgery can yield good outcomes. We present a case of a 57-year old gentleman with metastatic melanoma and cardiac involvement who initially presented as a ventricular arrhythmia and was successfully treated with immune checkpoint inhibitors and targeted therapy.

## Introduction

In the United States in 2019, there were an estimated 96,480 newly diagnosed cases of melanoma, comprising 5.5% of all newly diagnosed cancer cases. Four percent of the patients were expected to have distant metastasis at presentation. The five-year relative survival of metastatic melanoma was estimated at 24.8% [1]. Melanoma is highly aggressive with unpredictable biological behavior and can metastasize to any organ, with the most frequent sites being liver, bone, and brain [2]. In stage IV disease, the site of metastasis and the level of lactate dehydrogenase are the important prognostic factors of survival [3]. The heart is frequently involved in metastatic melanoma. According to a study of 70 autopsy cases of patients with melanoma, cardiac involvement was found in 45 of the cases; however, cardiac involvement was identified in less than 2% of the patients while they were still alive [4,5]. Cardiac metastasis can cause multiple complications such as right ventricular obstruction, arrhythmia, heart failure, and pericardial effusion. We describe a case of a 57-year-old male with metastatic melanoma to heart who initially presented with ventricular tachycardia.

## Case presentation

A 57-year-old Caucasian male presented to the emergency department with two episodes of chest discomfort, palpitations, and shortness of breath. Past medical history was significant for melanoma of the right shoulder without metastatic spread, diagnosed six years prior to his current presentation. The tumor was classified as a *superficial spreading* subtype, Clark IV, for which the patient underwent resection. No recurrence had been noted. Differential diagnosis was broad and included acute coronary syndrome, acute pericarditis, acute heart failure exacerbation, and the involvement of tumor to the heart.

**Figure 1:**
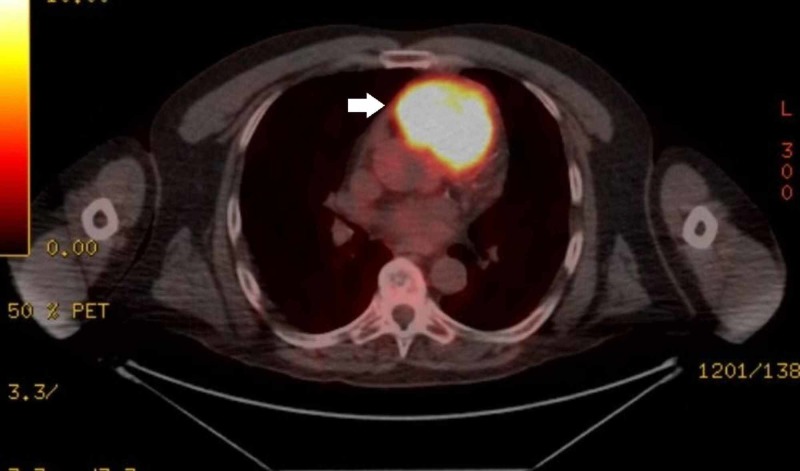
F-fluorodeoxyglucose-positron emission tomography (FDG-PET) revealing an intensely hypermetabolic soft tissue mass within the right ventricle (arrow) measuring 6.9 x 6.2 cm.

**Figure 2:**
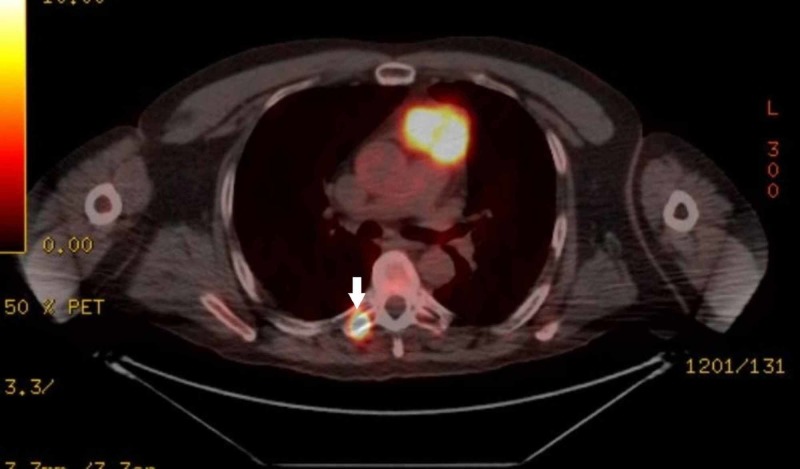
F-fluorodeoxyglucose (FDG) avid lesion within the lateral right T6 transverse process (arrow).

His electrocardiogram (ECG) revealed episodic non-sustained ventricular tachycardia, for which he was treated with amiodarone. A transthoracic echocardiogram showed a 6.4 x 3.7 cm, large heterogeneous mass, extending from the basal free wall of the right ventricle into the ventricular outflow tract, with mild right ventricular hypokinesis and dilation. Computed tomography (CT) imaging revealed a cardiac mass extending into the pulmonary trunk with stenosis of the proximal pulmonary truck. Subsequently, the patient received a whole-body positron emission tomography-CT (PET-CT) scan, revealing an intensely hypermetabolic avid soft tissue mass within the right ventricle, extending into the pulmonary trunk (Figure 1), and a hypermetabolic focus within the transverse process of T6 (Figure 2), consistent with metastatic disease.

A CT-guided percutaneous needle biopsy was performed, which was consistent with atypical spindle cells. Immunohistochemical stains demonstrated expression of vimentin, caldesmon, smooth muscle actin, S100, and CD31, without expression of desmin, myogenin, pankeratin, CD34, HMB45, or MART1. The Ki-67 proliferation index was high (approximately 20%). A diagnosis of high-grade intimal sarcoma with smooth muscle differentiation was made. The patient was initially started on doxorubicin and ifosfamide and underwent four cycles with a follow-up PET scan showing progression of metastatic disease. The patient was referred to our institute for failure of response and further management of his metastatic disease.

His case was discussed at the multidisciplinary sarcoma tumor board. He underwent a complete resection of the mass with a reconstruction of the right ventricular free wall and right ventricular outflow tract with a bovine pericardial patch with no complications.

**Figure 3:**
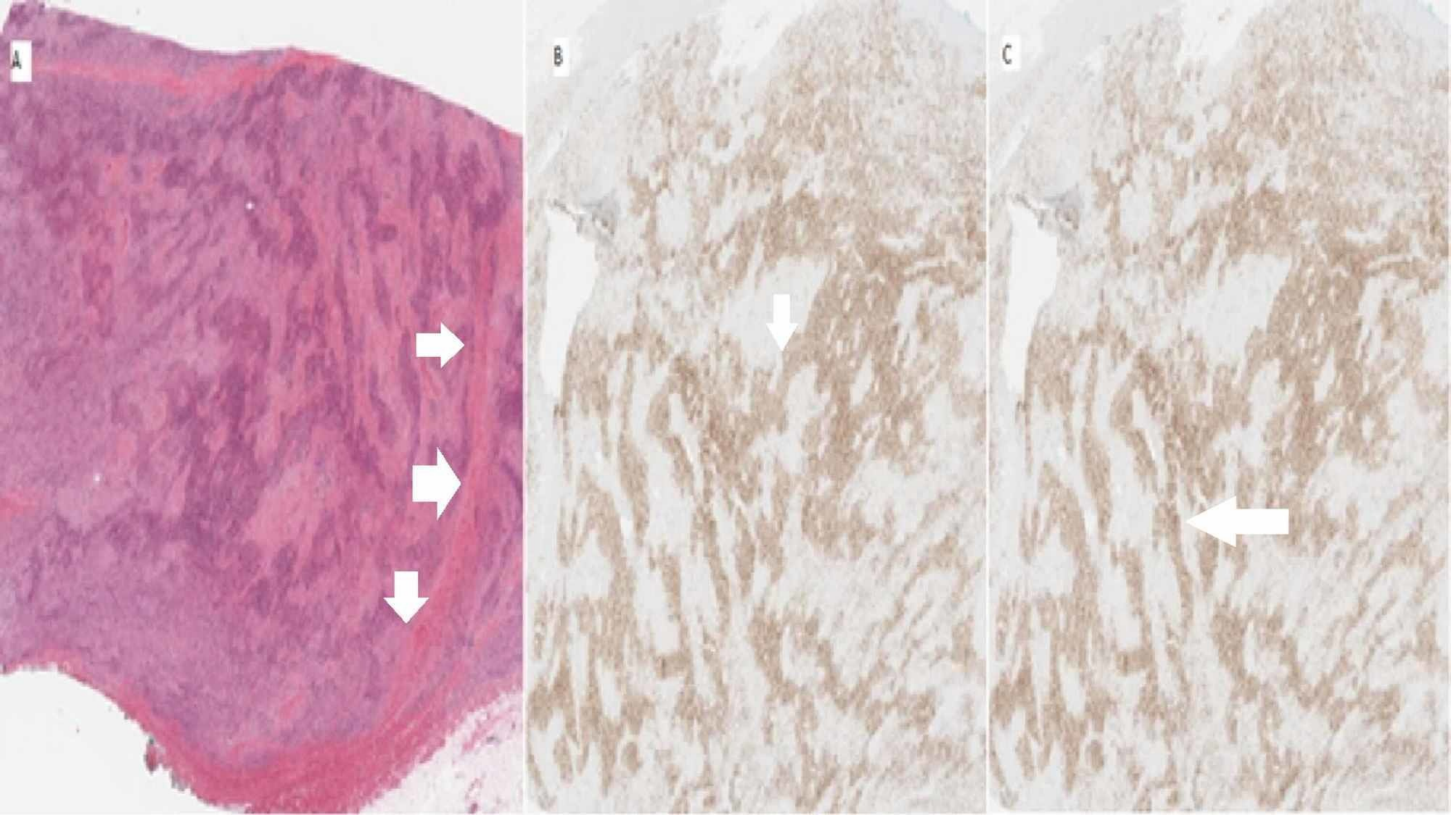
Histology showing H&E stain (A) demonstrating malignant spindle cell neoplasm infiltrating cardiac muscle with extension into pericardium; (B) immunohistochemical staining positive for S-100 (both nuclear and cytoplasmic); (C) immunohistochemical staining positive for SOX-10.

The pathology report was significant for a yellow to white firm mass measuring 8.5 x 7.0 x 4.0 cm. This showed malignant pleomorphic spindle cell neoplasm infiltrating cardiac muscle and pericardium. Immunohistochemical staining demonstrated that the neoplasm was diffusely and strongly positive for S-100 (both nuclear and cytoplasmic) and SOX-10 (nuclear); however, it was negative for AE1/3, CAM 5.2, desmin, CD34, SMA, and MDM2 (Figure 3). Given the patient’s previous history of melanoma, these findings were supportive of a diagnosis of metastatic malignant melanoma and against a diagnosis of intimal sarcoma.

**Figure 4:**
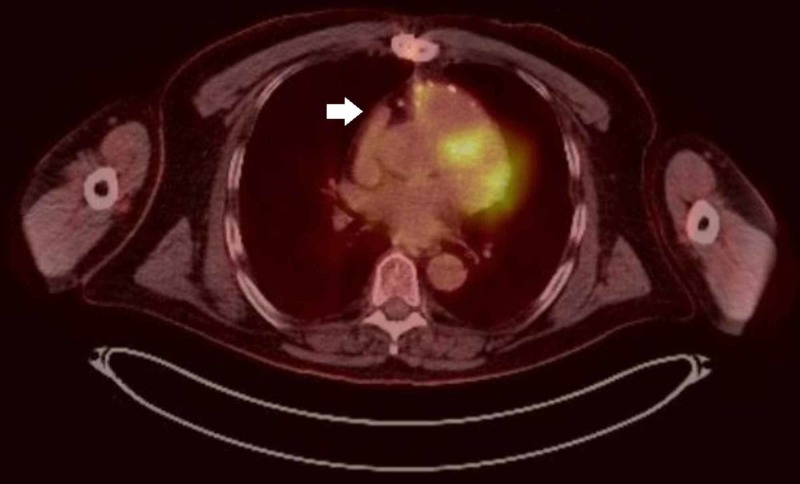
Post-treatment changes in the right ventricular wall without discrete F-fluorodeoxyglucose (FDG) avid mass (arrow). No hypermetabolic chest mass, fluid collection, or lymphadenopathy.

**Figure 5:**
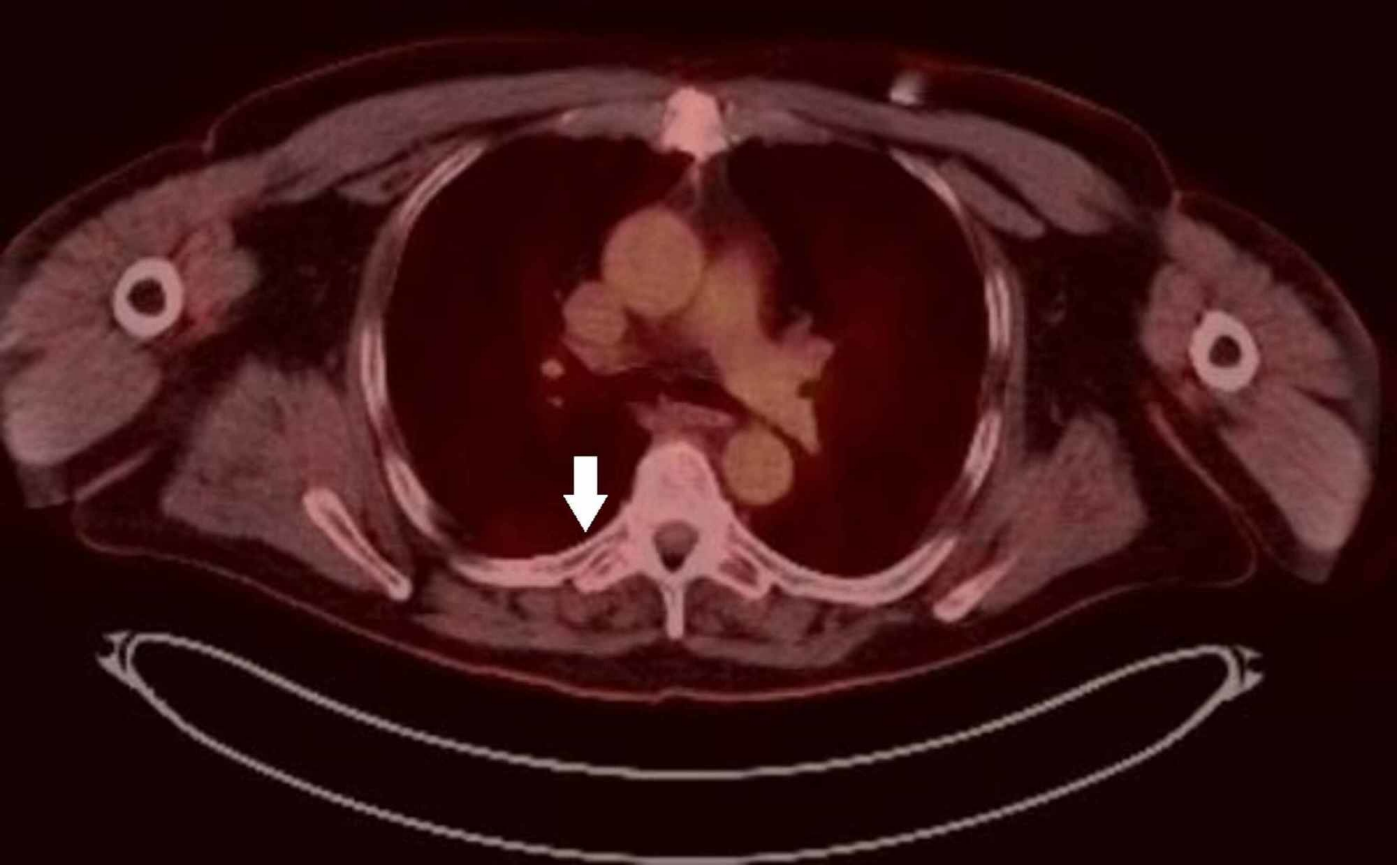
Interval resolution of increased F-fluorodeoxyglucose (FDG) uptake within the T6 right transverse process (arrow) and left scapular angle, compatible with treated metastatic lesions.

The patient began combined treatment of immunotherapy with ipilimumab and nivolumab. After four cycles, follow-up PET showed completed resolution of metastatic disease (Figures 4, 5), and the regimen was changed to maintenance nivolumab.

## Discussion

In the last decade, with the emergence of immune checkpoint inhibitors and BRAF- and MEK-targeted therapies, the survival outcomes of metastatic melanoma have significantly improved [6]. Nearly one-third of melanoma patients will develop metastasis with most common sites being lungs, liver, brain, and bone [7].

Metastatic disease to the heart is more common than primary cardiac tumors [8]. Malignant melanoma is a highly aggressive tumor with an unpredictable biological behavior. It frequently metastasizes to the heart, most frequently affecting the right atrium. It is believed to spread hematogenously to the heart and mostly involves the pericardium and myocardium [5,9]. Cardiac involvement can be indolent and go unnoticed for a long time. Antemortem diagnosis is rare as patients remain asymptomatic.

To our knowledge, reports of patients who present with cardiac arrhythmias as a manifestation of cardiac metastatic melanoma are sparse [10,11]. The exact mechanism of how an intracardiac lesion can lead to ventricular tachycardia is unclear. The myocardial insertion of the tumor may cause heterogeneities in the electrophysiological properties of myocardial tissue, thus stimulating the initiation of ventricular tachycardia through a single-reentrant focus [12].

Regardless of the exact mechanism of the tachycardia, this case reinforces the importance of performing a detailed physical examination and subsequent investigations to exclude cardiac metastasis in patients with melanoma. Diagnosis is usually made with transthoracic and transesophageal echocardiograms, magnetic resonance imaging (MRI), and CT scan [13].

In the past, the prognosis of metastatic melanoma was poor. However, with new treatment modalities and improved survival outcomes of advanced melanoma, the prevalence of the disease has increased [14]. Our patient underwent a successful resection of the cardiac melanoma and had complete resolution of metastatic disease after treatment with the immune checkpoint inhibitors, ipilimumab and nivolumab.

## Conclusions

We describe a case of stage IV melanoma with cardiac and bone metastasis, presenting as ventricular tachycardia. The patient had an excellent response to surgical resection of the cardiac mass, followed by combination immunotherapy. Prior to the advent of contemporary therapies, cardiac metastases implied a very poor prognosis. The use of immune check point inhibitors and targeted therapy has greatly improved survival outcomes of metastatic melanoma. Aggressive therapy of cardiac metastasis including cardiac surgery can yield good outcomes.
